# Extracellular Vesicles as Diagnostics and Therapeutics for Structural Epilepsies

**DOI:** 10.3390/ijms20061259

**Published:** 2019-03-13

**Authors:** Jenni Karttunen, Mette Heiskanen, Anssi Lipponen, David Poulsen, Asla Pitkänen

**Affiliations:** 1 A. I. Virtanen Institute for Molecular Sciences, University of Eastern Finland, P.O. Box 1627, FI-70211 Kuopio, Finland; jmk78@cam.ac.uk (J.K.); mette.heiskanen@uef.fi (M.H.); anssi.lipponen@uef.fi (A.L.); 2 University at Buffalo, Jacobs School of Medicine and Biomedical Sciences, Clinical and Translational Research Center (CTRC), Department of Neurosurgery, Buffalo, NY 14203, USA; davidpou@buffalo.edu

**Keywords:** antiepileptic drug, anti-seizure drug, biomarker, epileptogenesis, post-traumatic epilepsy, traumatic brain injury

## Abstract

Extracellular vesicles (EVs) are small vesicles involved in intercellular communication. Data is emerging that EVs and their cargo have potential as diagnostic biomarkers and treatments for brain diseases, including traumatic brain injury and epilepsy. Here, we summarize the current knowledge regarding changes in EV numbers and cargo in status epilepticus (SE) and traumatic brain injury (TBI), which are clinically significant etiologies for acquired epileptogenesis in animals and humans. We also review encouraging data, which suggests that EVs secreted by stem cells may serve as recovery-enhancing treatments for SE and TBI. Using Gene Set Enrichment Analysis, we show that brain EV-related transcripts are positively enriched in rodent models of epileptogenesis and epilepsy, and altered in response to anti-seizure drugs. These data suggest that EVs show promise as biomarkers, treatments and drug targets for epilepsy. In parallel to gathering conceptual knowledge, analytics platforms for the isolation and analysis of EV contents need to be further developed.

## 1. Introduction

According to the World Health organization, more than 50 million people worldwide have epilepsy (https://www.who.int/news-room/fact-sheets/detail/epilepsy, accessed on: 10 February 2019). Each year, 2.2 million patients are newly diagnosed with epilepsy. Thus, a new epilepsy diagnosis occurs every 13 sec. Despite the fact that more than 20 anti-seizure drugs have been approved for clinical use, seizures for about 30% of epilepsy patients remain uncontrolled [1]. Although more than 50 studies have demonstrated preclinical efficacy for preventing epileptogenesis after experimental brain injury, no therapeutic agents have progressed to the clinic [2]. One of the major factors hindering the development of anti-epilepsy treatments has been the lack of biomarkers that can stratify patients at highest risk of developing epilepsy after brain insult. Without such stratifying biomarkers, the length of time and the numbers of patients required to perform well-powered clinical antiepileptogenesis studies becomes unreasonable [3]. However, recent observations suggest that the contents of extracellular vesicles (EVs), assessed in liquid biopsy, can serve as prognostic biomarkers for the progression of brain diseases, and raise hope that EVs may be useful as a biomarker source for epilepsy.

**Figure 1:**
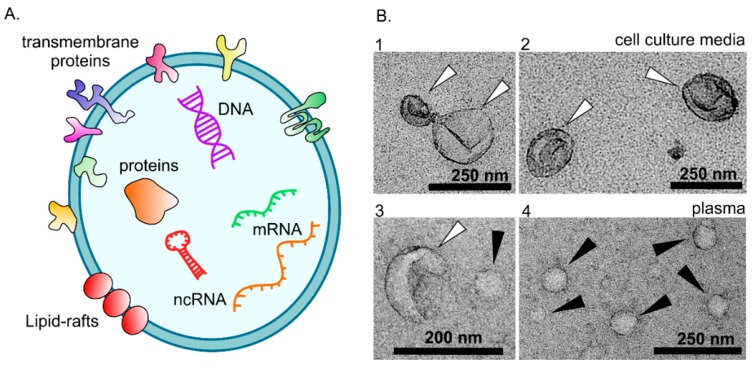
(**A**) A schematic presentation of the structure of extracellular vesicles (EVs). A lipid bilayer encapsulates the water-soluble cargo. EVs can carry a variety of regulatory molecules, including DNAs, messenger RNAs (mRNA), noncoding RNAs (ncRNA), proteins, and enzymes. (**B**) Transmission electron microscope images of EVs (white arrowheads) isolated from mesenchymal stem cell culture media (**B_1_** and **B_2_**) and rat plasma (**B**_3_ and **B_4_**). The cup-shaped form is caused by the collapse of the water-containing vesicle during the sample preparation. Plasma lipoproteins (black arrowheads in **B**_3_ and **B_4_**) show round morphology.

EVs are small membrane vesicles that neuronal and non-neuronal cells secrete to the extracellular environment and are involved in intercellular communication (Figure 1). EVs were initially described in 1967 when Wolf reported that platelets secrete “cell dust” [4]. This finding remained in the background until 2006 and 2007 when it was realized that EVs encompass both mRNA and miRNA. Moreover, this observation became even more intriguing when it was determined that target cells were able to translate EV-associated mRNAs into proteins [5,6]. Since these pivotal observations, it has been shown that EVs also carry DNA, proteins and enzymes involved in lipid metabolism [7,8,9,10,11]. During the last decade, a growing number of research articles have been published which emphasize the role of EVs in the pathophysiology of cancer and neurodegenerative diseases [12,13,14].

The fundamental role of EVs in intercellular communication offers many possibilities for their use in the diagnosis and treatment of diseases. The secreted EV cargo can reflect the functional status of their cells of origin. Consequently, the analysis of EV cargo together with their cell-type specific surface proteins makes EVs attractive targets to be profiled from a “liquid biopsy” in order to probe disease pathologies rather than tissue biopsy [15]. This is particularly true for brain diseases, in which the availability of brain tissue is rarely possible.

Mesenchymal stem cell-secreted EVs have shown great promise as therapeutic agents for various diseases, including myocardial ischemia, ischemic stroke, and chronic kidney disease [16,17,18]. Many ongoing studies aim to modify EV cargos with the goal of increasing therapeutic potential [19,20]. An alternate approach has been to modify the structure of EV surface peptides to make them more permeable to the blood-brain-barrier and target specific cell types to treat neurological diseases [21].

Unfortunately, the nomenclature relating to vesicles has been diverse. Extracellular vesicle was a term established in 2009 to describe all membrane-derived vesicles secreted by cells into the extracellular space. Based on their origin, EVs can be divided into subgroups including: (1) exosomes, that have an endosomal origin (30–150 nm); (2) microvesicles (also called ectosomes), that bud from the cellular outer membrane (100–1000 nm); and (3) apoptotic bodies that are released during apoptosis (50–5000 nm) [22,23]. The term “microparticles” has also been used in several studies. Here, we refer to all vesicles as EVs.

In this review, we address the current methodological limitations that should be considered when performing EV-related studies and interpreting their results. We summarize the current knowledge of the role of extracellular vesicles in epilepsy and epileptogenesis with a focus on post-traumatic epileptogenesis (PTEgenesis). Finally, we report our in silico analysis of changes to EV-related transcripts after TBI and SE in brain tissue in relation to anti-seizure drug treatment.

## 2. Methodological Challenges in EV Isolation and Cargo Analysis in Liquid Biopsies

The interest for EVs has grown rapidly and the methods used in EV research are constantly evolving. The mission of the International Society for Extracellular Vesicles (ISEV) is to advance extracellular vesicle research globally. In 2014, ISEV determined and published guidelines for the minimal requirements for the analysis of EVs (MISEV) [24]. The guidelines were further improved using contributions from over 380 ISEV members in 2018 (MISEV2018) [25,26]. In addition, an open-source knowledgebase EV-TRACK (http://evtrack.org/, accessed on: 15 January 2019) was introduced in 2017. EV-TRACK is a database for EV-related studies and its aim is to centralize experimental parameters of the rapidly expanding EV-related research [27]. 

Sequential ultracentrifugation, density gradient centrifugation, size-exclusion chromatography, and various precipitation-based methodologies applying different reagents have all been used for the isolation of EVs [28]. Each of these methods have their respective limitations, which should be noted when they are applied [22]. In addition, different source materials have unique properties that should be considered when the isolation method is selected. This applies especially to body fluids that are used in biomarker discovery.

When isolating EVs from plasma or serum, lipoproteins are one of the major sources of contamination. The number of EVs is only a tiny fraction of the number of lipoproteins in plasma. Simonsen (2017) estimated that 1 mL of plasma contains 10^7^ to 10^9^ EVs but 10^16^ lipoprotein particles [29]. Unfortunately, subgroups of lipoproteins overlap either in size or density with EVs, which complicates the isolation process [29,30]. Lipoproteins co-purify with EVs in differential ultracentrifugation, density gradient purifications and in precipitation-based purifications [31,32]. In addition, the commonly used techniques to measure EV concentration and size distribution [nanoparticle tracking analysis (NTA) and tunable resistive pulse sensing (TRPS)] do not distinguish EVs from lipoproteins or larger protein aggregates [33,34,35]. Recently, Karimi et al. combined density-based separation to size-exclusion chromatography and were able to isolate cup-shaped EVs from 6 mL of human plasma. The protein profile differed from earlier plasma-EV studies which used other purification methods, but resembled the proteomics profile of EVs isolated from cell culture media [30].

Recently, sucrose-density gradient, sucrose-cushion, and iodixanol floatation density gradient methods have been used to isolate EVs from brain tissue [36,37,38]. When isolating tissue EVs, one challenge relates to collection of vesicles from the extracellular space without the disruption of cells during tissue harvesting, processing, or freezing. In EV isolation from the brain tissue, common contaminants include intracellular and synaptic vesicles [26,39].

microRNAs and other circulating small RNAs are common targets for biomarker discovery from liquid biopsy for different indications [3,40]. Most circulating miRNAs are apparently carried by the Argonaute protein complexes [41,42], and several articles have reported the presence of small RNA also in lipoproteins [43,44,45]. However, EVs also carry small non-coding RNAs. One size-exclusion chromatography-based study estimated that EVs carry up to 15% of plasma miRNAs [42]. 

When examining the miRNA cargo of EVs in plasma, it is important to remove other potential carriers of miRNA during EV-purification. Recently, we evaluated precipitation-based purification methods and showed that vesicle-free miRNAs are found in the purified EV-pellet [32]. Van Deun et al. detected positive Western blot signal with Ago-2 antibody from EVs purified by precipitation-based methods and ultracentrifugation [46]. Multiple studies have compared EV isolation methods and shown that the method used does have an effect on the miRNome of EVs preparations [47,48,49]. 

In December 2017, ISEV organized a workshop on “extracellular vesicles as disease biomarkers”. The summary published in the Journal of Extracellular Vesicles highlighted gaps in current knowledge related to pre-analytical issues affecting EV purification and the need to use standardized isolation and analysis methods including the use of the EV-TRACK and MISEV [50].

## 3. Heterogeneity of EVs

One cell type can secrete several populations of vesicles carrying RNA. Lässer et al. studied one cell line and found extracellular RNA in two density fractions with very distinct RNA and protein profiles [51]. For example, colorectal cancer cells secrete at least three different types of vesicles: two subpopulations of exosomes and shed microvesicles. All three populations have distinct profiles of proteins, miRNA, mRNA, and long noncoding RNAs [52,53]. The situation is even more complex with plasma and serum samples, because the vesicles originate from multiple organs and cell types. 

Currently, there are no comprehensive studies that define the origins of plasma EVs. This is particularly true for brain-derived EVs. To obtain a general idea of the origin of plasma vesicles, Arraud et al. used cryo-transmission electron microscopy combined with gold-immunolabelling and cell-type-specific antibodies. These authors observed spherical, tubular, and large membrane particles in the EV fraction and estimated that more than 50% of plasma EVs originate from platelets (CD41-positive EVs) or erythrocytes (CD235a-positive EVs) [54]. In another study, Mustapic et al. used the ExoQuick^®^ reagent to precipitate EVs and reported that L1-CAM specific (i.e., presumed to originate from neurons) vesicles comprised only 5–10% of the total number of plasma EVs [55]. 

The search of brain-cell specific EV markers is ongoing. L1-CAM and GluR2 are found in the EVs released by cultured cortical neurons, and consequently, they have been used as neuron-specific EV-markers [55,56,57,58]. Still, according to the Human Protein Atlas (www.proteinatlas.org, accessed on: 15 January 2019), L1-CAM is expressed also in the kidney, but its presence in kidney-derived plasma EVs is not clear.

## 4. Extracellular Vesicle Profile in Plasma and CSF during the Epileptogenic Process

The secretion of EVs into body fluids is altered in several diseases, including neurodegenerative diseases (for reviews, see References [14,59]). Consequently, both the number and the cargo of EVs in liquid-biopsy are considered as potential biomarkers to monitor the progression of the molecular and cellular processes, leading to brain diseases such as epilepsy, and can also be used to monitor a therapeutic response [15]. 

Based on recent definitions, epileptogenesis refers to the development and extension of tissue capable of generating spontaneous seizures, resulting in (a) development of an epileptic condition and/or (b) progression of the epilepsy after it is established [2]. Importantly, the epileptogenic process continues even after the occurrence of the first seizure, which make the initiation of clinical antiepileptogenesis treatment trials feasible even after the epilepsy diagnosis. An epileptic seizure refers to a transient occurrence of signs and/or symptoms due to abnormal excessive or synchronous neuronal activity in the brain [60]. Epilepsy is a disease of the brain defined by any of the following conditions: (a) at least two unprovoked (or reflex) seizures occurring >24 h apart, (b) one unprovoked (or reflex) seizure and a probability of further seizures similar to the general recurrence risk (at least 60%) after two unprovoked seizures, occurring over the next 10 years, and (c) diagnosis of an epilepsy syndrome [61].

To our knowledge, four articles have analyzed EVs in epilepsy. Huttner et al. quantified the amount of somatic cell marker CD133 on membrane particles in the CSF of partial epilepsy patients. They found increased levels of CD133 on membrane particles and suggested that it may indicate increased membrane shedding [62]. However, these authors only quantified CD133 levels and not particles. Therefore, it is possible that the number of particles did not change but the particles contained more CD133. Raoof et al. analyzed EV-miRNAs from CSF of temporal lobe epilepsy (TLE) and status epilepticus (SE) patients [63]. In a second article, Raoof et al. analyzed EV-miRNAs from the plasma of TLE patients [64]. Yan et al. analyzed EV-miRNA profile from the plasma of patients with mesial temporal lobe epilepsy with hippocampal sclerosis [65]. These articles focused solely on the miRNA contents and did not characterize or quantify EVs. The findings of these studies are discussed in more detail later in this review. Epilepsy is, however, the end stage of the epileptogenic process. To understand the potential dynamics of EVs during the epileptogenic process, we summarize the data relative to EV vesicles in body fluids after TBI, a major etiology for structural epilepsies.

## 5. EV number in Plasma and CSF during Epileptogenesis after TBI

Currently, there are no prognostic biomarkers to identify subjects who have a high risk of epileptogenesis after TBI [3]. We will next review the studies which have made attempts to use EVs as a source for biomarker discovery in TBI as it is likely that some of those biomarkers could also have value as prognostic biomarkers for post-TBI epileptogenesis.

### 5.1. Experimental TBI

#### 5.1.1. CCI 

EV numbers increase in the CCI-induced mouse models of TBI [66,67,68]. Andrews et al. reported that the number of occludin-positive mouse plasma EVs increased 24 h after CCI-induced TBI. Occludin is a protein located in the tight junctions between the endothelial cells in the BBB. Andrews et al. suggested that the secretion of such EVs is an indication of vascular remodeling. [66]. Kumar et al. (2017) used flow cytometry to measure the total number of blood EVs (300–1000 nm) positive for microvesicle surface marker Annexin V in CCI-induced TBI mice 24 h post-injury [67]. They found an increase in the number of annexin V+ vesicles in TBI animals relative to uninjured controls. In addition, these authors observed an increase in the number of microglial-derived vesicles (P2Y12+/CD45+) in TBI animals, suggesting that TBI causes a release of microglial EVs from the brain into the circulation. In another study, Hazelton et al. found that the plasma EV concentration in mice with CCI was highest at 24 h post-TBI [68]. In addition, Hazelton et al. reported that the mean diameter of EVs decreased at 24 h (94.33 ± 2.404 nm) compared to earlier time points (101.67 ± 7.12 nm at 6 h, 102.66±2.88 nm at 12 h).

#### 5.1.2. Lateral FPI

Bohman et al. measured the EV number in the serum of fluid-percussion injury (FPI)-induced piglets [69]. They found that EV numbers increased at 1 h after injury compared to baseline. In addition, Chen et al. (2018) studied EV release in the brain tissue of rats following FPI. They found an increase in the expression of the EV marker proteins CD63 and CD81, in the hippocampus at 6 h after injury. They concluded that the increased EV release was connected to the activity of connexin 43, a gap junction protein in astrocytes, as blocking the activity of connexin 43 resulted in a reduction of hippocampal EV numbers after TBI [70].

#### 5.1.3. Other Models

In contrast to the above studies, Midura et al. reported that the total numbers of EVs in mouse plasma decreased at 3–24 h after weight-drop-induced TBI, but the number returned to the control levels by 3 d post-TBI [71]. 

### 5.2. Human TBI

#### 5.2.1. Plasma

Several articles have addressed the changes in EV numbers within plasma and CSF of patients with TBI. Morel et al. observed an increase in the number of EVs in human plasma at the acute phase (day 0) of severe TBI. During the following days, EV numbers decreased progressively and reached the control level by day 10 [72]. Nekludov et al. reported comparable findings by showing that the levels of platelet, endothelial, and leukocyte-derived EVs (recognized based on the presence of markers CD42b, CD144 and CD45, respectively) increased acutely at 1–72 h after TBI [73]. EV numbers were highest in the samples collected in the emergency room, and the number decreased sharply by 6 h. During the period of 6–72 h after injury, EV numbers slowly decreased but remained slightly higher than controls.

#### 5.2.2. CSF

EV numbers have also been reported to increase in human CSF after TBI. Morel et al. observed that the number of EVs in CSF was elevated at day 0 after TBI. The EV number returned to control levels by day 10 after TBI. Based on antibody-mediated capture, EVs were mainly derived from platelets (GPIb+) and endothelial cells (CD31+), suggesting they represented blood-brain barrier (BBB) damage or injured blood vessels [72]. Patz et al. (2013) quantified particles in CSF by flow-cytometry and showed that the number of EVs were higher in the CSF of severe TBI patients compared to uninjured controls. However, the samples were not collected at standardized time points as the timing of sampling varied from 2 to 29 days after injury depending on the patient [74]. Manek et al., found a higher CSF EV concentration and smaller vesicles at 12 h post-injury in patients with TBI (mode 74–98 nm) compared to controls (mode 99–104 nm) [75]. Similarly, Kuharic et al. observed an increase in the number of EVs in CSF after TBI, peaking at 24 h post-injury. The modal size of EVs was larger on days 4–7 (205 ± 40 nm) than in controls (157 ± 24 nm) or on 1 or 2–3 days post-injury (158 ± 57 nm and 141±41 nm) [76].

### 5.3. Caveats Related to Analysis EVs after TBI

Although several studies showed changes in EV numbers after both experimental and clinical TBI, large variation in methodology used for the isolation and analysis of EVs should be noted (Table 1). Most of the studies used differential centrifugation, which separates particles by their size [77]. However, in some of these studies, the speed of the last centrifugation was rather low being ≤13,000 g, which can exclude the smallest EV subpopulation, since their isolation requires ultracentrifugation at ≥100,000 g (for a review see Reference [78]). In addition, not all studies analyzed EVs in accordance with the MISEV guidelines [24]. Several studies quantified EVs with flow cytometry or NTA but lacked further characterization (Table 1). Many of these studies were published before the release of MISEV guidelines, and the requirements of EV research were not widely known at that time. Thanks to methodological guidelines, more recent articles have characterized EVs more accurately.

Taken together, these studies show that the EV numbers in plasma and CSF change after experimental and clinical TBI. All but one study showed an increase in the number of EVs. Based on the studies, EV numbers in plasma and CSF appear to be highest during the first 24 h after the injury both in animal models and human patients. After 24 h, the EV numbers gradually return to normal levels. Interestingly, Midura et al. reported that the number of EVs was lowest during the first 24 h after the injury, which contradicts other studies. In addition to changes in EV numbers, some studies have reported changes in EV size. However, due to huge variation in the EV isolation and quantification methods, the studies mentioned in this review are not conclusive regarding the changes in EV number and size in CSF and plasma after TBI.

## 6. Regulation of Body-Fluid EV Cargo during Epileptogenic Process with Focus on microRNAs

Several studies have reported altered miRNA profiles in brain tissue and plasma after status epilepticus (SE) and TBI, and these findings have been comprehensively reviewed earlier [3,40,79]. EVs contain an interesting composition of circulating small RNAs. EV miRNA content might reveal new insights into the molecular mechanisms taking place in the brain and serve as pathology-specific mechanistic biomarkers. Until now, only a few studies have examined EV cargo in epilepsy or epileptogenesis.

### 6.1. Post-Traumatic Epileptogenesis

Harrison et al. isolated EVs from the brains of CCI-injured mice at 7 d post-TBI and pinpointed five differentially expressed miRNAs, of which miR-21 had the highest increase compared to controls [80]. Circular RNAs (circRNAs) have been hypothesized to be sponges that remove miRNA. Zhao et al. found 231 differentially expressed circRNAs within brain extracellular EVs in FPI-injured mice at 3 h post injury [81]. 

In 2013, Patz et al. performed a miRNA array analysis of EVs isolated from the CSF of severe TBI patients. They found 81 mature miRNAs of which miR-9 and miR-451 were the only ones differentially expressed compared to controls [74]. An interesting recent study introduced a miRNA-based biomarker panel to diagnose TBI, both in a mouse model and in human patients [58]. They exposed mice to blast overexposure injury and isolated brain-derived (GluR2-positive) EVs from plasma using microchip diagnostics (Track-Etched magnetic NanoPOre, TEMPO). RNA sequencing and machine learning processing were used to generate a biomarker panel consisting of seven miRNAs (miR-129-5p, miR-212-5p, miR-9-5p, miR-152-5p, miR-21, miR-374b-5p, and miR-664-3p). As a result, they were able to differentiate TBI patients from healthy controls with high accuracy (AUC= 0.9) [58]. 

### 6.2. Epilepsy

Yan et al. studied patients with mesial temporal lobe epilepsy with hippocampal sclerosis (mTLE-HS) and found 50 differentially expressed miRNAs in plasma EVs. Two miRNAs were upregulated and 48 miRNAs were downregulated [65]. 

Raoof et al. performed miRNA array analysis of CSF samples from patients with TLE or SE. They isolated EV-related and argonaute-2–related miRNAs from pooled CSF samples and measured the top hits, using microarray and quantitative PCR. They found unique distribution patterns for different miRNAs between EVs and Argonaute2. For example, miR-19b-3p was found in the EV fraction in patients with multiple sclerosis that served as controls to epilepsy patients. In TLE and SE patients, miR-19b-3p was bound to argonaute-2 [63]. The same strategy was used in a later study, that profiled plasma biomarkers for adult TLE [64]. Primary miRNA discovery was made from total plasma with microarray and sequencing. Later, EV isolation and argonaute-2 immunoprecipitation were used to determine the carrier of the three potential biomarker miRNAs: miR-27a-3p, miR-328-3p, and miR-654-3p. Authors reported a higher diagnostic accuracy in EV samples compared to argonaute immunoprecipitation [64]. One caveat to many of these studies is the use of a precipitation-based method in EV isolation (Table 2) as this method also co-precipitates vesicle-free miRNAs [32].

## 7. EV-Related Transcripts are Positively Enriched in Rodent Models of Epilepsy and Epileptogenesis

To investigate changes in EV-related gene expression in brain tissue, we analysed available transcriptomics data from rodent models of epileptogenesis and epilepsy. For the analysis of epileptogenesis (potential epilepsy inducing injury) models, we used microarray data from the hippocampus and cortex sampled at 32 h after lateral FPI [90] or 4 months after CCI-induced TBI [91]. For the analysis of epilepsy models, we used chronic data from the hippocampus after pilocarpine-induced epilepsy and amygdala stimulation-induced epilepsy [92]. Data were examined using gene set enrichment analysis (GSEA) [93,94]. A rank list was generated from the microarray or mRNA sequencing data. A gene set for the GSEA consisted of the Top100 EV-related proteins from Vesiclepedia (http://microvesicles.org/, accessed on: 1 December 2018) [95]. Enrichment was considered significant when the false discovery rate (FDR) was less than 0.05. In GSEA, significant positive enrichment indicates that the expression of the tested genes as a group is increased. Significant negative enrichment indicates decreased expression.

### 7.1. Post-Traumatic Epileptogenesis

**Figure 2:**
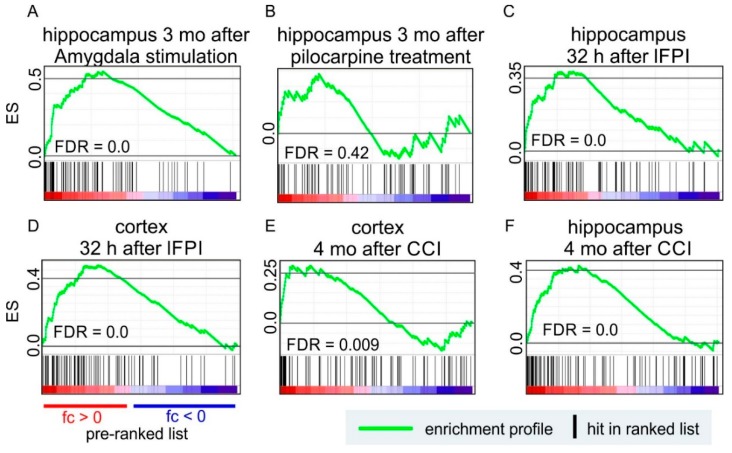
The effect of epileptogenesis and epilepsy on EV-related genes in four rodent models of epileptogenesis: (i) status epilepticus (SE) triggered by electrical stimulation of the amygdala [92]; (ii) SE induced by intraperitoneal injection of a chemoconvulsant, pilocarpine [92]; (iii) traumatic brain injury (TBI) induced by lateral fluid-percussion [90]; and (iv) TBI induced with controlled cortical injury (CCI) in mice [91]. Gene expression data of the hippocampus or cortex was used to construct the rank list in Gene Set Enrichment Analysis. Top100 EV-related proteins from Vesiclepedia (http://microvesicles.org/, accessed on: 1 December 2018) were used to construct a gene set. The EV related genes were (**A**) positively enriched at 3 months (mo) in the amygdala stimulation SE-model, whereas (**B**) no enrichment was observed at 3 months in the pilocarpine SE-model. In the two TBI models of epileptogenesis, EV-related genes were positively enriched in the hippocampus and cortex at both (**C**,**D**) the acute (32 h post- TBI) and (**E**,**F**) chronic (4 months post-TBI) time point. Abbreviations: ES, enrichment score; FDR, false discovery rate; fc, fold change.

EV-related gene expression was positively enriched in the ipsilateral cortex and hippocampus sampled at 32 h after lateral FPI (Figure 2C,D). This correlated with previous findings, reporting an increase of number of EVs in CSF and plasma acutely after TBI. EV-related genes were also positively enriched at the chronic time point (4 months) after TBI, in both the cortex and hippocampus (Figure 2E,F). 

### 7.2. Epilepsy

Positive enrichment of EV-related genes was also observed in the hippocampus 3 months after amygdala stimulation-induced epilepsy (Figure 2A). No enrichment was observed at the chronic time-point (3 months) after pilocarpine-induced epilepsy (Figure 2B). 

These data indicate that EV-related gene expression in the brain is increased acutely in the FPI model of TBI as well as chronically in the amygdala stimulation model of epilepsy and the CCI model of TBI. These changes could be involved in epileptogenesis and these preliminary observations encourage further exploration of the role of EVs in epilepsy.

## 8. EV Therapy Improves the Functional Outcome after Status Epilepticus and TBI

Studies applying mesenchymal stem cell (MSC) as a therapy for neurological diseases (reviewed in [96]) including status epilepticus [97,98,99,100,101] and TBI [102,103,104] have provided encouraging favorable results. Further, it has been shown that beneficial effects can occur through the secretome of the cells [105,106]. As EVs are involved in intercellular signaling, they are one potential mediator for the beneficial effects. EV therapy has several advantages compared to stem cell treatments. EVs do not have a nucleus and they cannot self-replicate. They can be sterilized by filtration and they tolerate storage at -80 °C. The scale up, production, and purification of EVs may prove to be less problematic compared to the production of cell products [16].

### 8.1. Post-Traumatic Epileptogenesis

The beneficial role of EV therapy after TBI has been reported in several studies (Table 3). Zhang et al. administered MSC-EVs to rats after CCI-induced TBI. The treatment enhanced spatial learning, promoted sensorimotor functional recovery, increased vascular density and angiogenesis, increased neurogenesis in dentate gyrus, and finally, reduced brain inflammation. However, cortical lesion volume was not altered [82]. In a subsequent study, these same authors showed in 2016 that the EVs from MSCs cultured in 3D conditions showed better outcome, at least in spatial learning, compared to 2D cultured cells [83]. Later, Li et al. reported that EVs from stem cells obtained from human exfoliated deciduous teeth improved rat motor functional recovery and reduced neuroinflammation after TBI [84]. In 2016, Kim et al. first optimized MSCs to produce anti-inflammatory EVs. They then demonstrated that MSC-EV treatment decreased the level of proinflammatory cytokine IL-1β in mouse brain in a dose-dependent manner post-TBI. In addition, they showed improved cognitive function 1-month post-TBI. Beneficial effects were detected in the pattern separation test and the water-maze test [85]. Recently, Williams et al. used a swine model of TBI and showed that MSC-EV-treated animals had a significantly lower neurological severity score (NSS) during the first 5 days post-TBI as compared to controls. The animals also recovered faster (NSS = 0) [86]. 

More detailed analysis of the mechanism of EV therapy in TBI has been described recently. Gao et al. showed that endothelial colony-forming cell (ECFC)-derived EVs improved the neurological functional recovery after CCI in mice [87]. In particular, EV treatment restored the TBI-induced degradation of the tight-junction proteins, occludin and ZO-1. Further, EV-treated brains had a significant reduction in Evans blue dye extravasation and reduced brain edema. Thus, ECFC-EVs seemed to restore the BBB continuity through tight-junction proteins [87]. Patel et al. showed that long noncoding RNA MALAT1 is an important factor for the beneficial effect of adipose-derived stem cell (hASC) EVs after CCI-induced TBI with rats [88]. They analyzed the brain and spleen transcriptome and showed a MALAT1-dependent regulation of genes involved in inflammation and immune response. They also showed that the EVs migrate to the spleen, liver and lungs within 1 h after transplantation. The impact site in the brain was reached at 3 days post-TBI [88]. They propose that one possible mechanism could be the EV-induced inhibition on the release of peripheral macrophages and monocytes from the spleen. Consequently, a lower number of these immune cells will enter the brain through damaged BBB, which decreases secondary injuries. In 2016, Pischiutta et al. at first described the beneficial effects of human amniotic MSC administration in mice with CCI-induced TBI. Following, they used cortical slices that were exposed to oxygen-glucose deprivation and showed that the beneficial effect was not affected by destruction of the protein secondary structure with heat. They performed membrane filtration for cell culture media and narrowed down the beneficial effect on small metabolites [106].

The knowledge on EV isolation from cell culture media and the commonly used analysis methods are constantly developing. Dominkus et al. reported that when using ultracentrifugation purification, commonly used fluorescent PKH26 dye forms nanoparticles that resemble EVs. Particles are also capable of internalizing at least to primary astrocytes in a similar manner as EVs. Therefore, they can cause false positive results unless proper negative controls are included in the experiments [107]. The addition of fetal bovine serum (FBS) to cell culture media can also cause contamination of the purified EV fraction. Wei et al. showed in 2016 that FBS contains a large variety of RNAs that remain after vesicle-depletion centrifugation. They compared the results to 13 earlier exosomal RNA samples from public databases and showed that 2.6–17.2% of the reads corresponded to bovine-specific transcripts indicating FBS-derived contamination [108]. Later, a review by Tosar et al. continued the analysis and addressed the question of ribonucleic artefacts in more detail [109]. Of the studies reviewed here, only Patel et al. used unconditioned media as a vehicle control and, in addition, conditioned media depleted of exosomes (TdCM) as a second control. Interestingly, TdCM showed minor improvement compared to the vehicle control. Still, the beneficial effect of EVs were evident [88].

### 8.2. Epilepsy

To our knowledge, only Long et al. have described the beneficial effects of EV therapy in epilepsy. They used MSC-EVs to treat pilocarpine-induced SE in mice. Intranasally-administered EVs reached the hippocampus within 6 h following administration. Long et al. found a decrease in proinflammatory signals and an increase in anti-inflammatory cytokines within the hippocampus at 24 h post-SE, reduced neuron loss 4d post-SE, promoted normal neurogenesis in the hippocampus, and preserved cognitive functions in the chronic phase [89]. These results suggest that the beneficial effects of MSC treatment can also be achieved with the EVs secreted by these cells.

## 9. Anti-Seizure Drugs Modulate the Expression of Genes Related to EVs

Treatment of epilepsy with anti-seizure drugs can last for tens of years, and one question is: do anti-seizure drugs affect EVs? So far, there is no literature available. To investigate whether anti-seizure drugs modify EV gene expression, we performed an in silico analysis using LINCS database, containing drug-induced transcriptomics profiles of cell lines to assess the possible effect of anti-seizure drugs on the expression of EV-related genes [110]. We found reproducible (golden) signatures (sig) for four anti-seizure drugs: carbamazepine (3 cell lines; CBZ-sig), primidone (2 cell lines; PRI-sig), gabapentin (1 cell line; GB-sig), and lamotrigine (1 cell line; LTG-sig), out of 27 drugs (acetazolamide, bromide, carbamazepine, clobazam, eslicarbazepine acetate, ethosuximide, felbamate, gabapentin, lacosamide, lamotrigine, levetiracetam, mesuximide, oxcarbazepine, pheneturide, phenobarbital, phenytoin, pregabalin, primidone, retigabine, rufinamide, stiripentol, sulthiame, tiagabine, topiramate, valproate, vigabatrin and zonisamide) when level 5 data was searched (GEO: GSE92742). The z-score (corresponding fold change to control) of the signatures was used as a rank list, and Top100 EV-related proteins from Vesiclepedia were used as a gene set in GSEA. 

**Figure 3:**
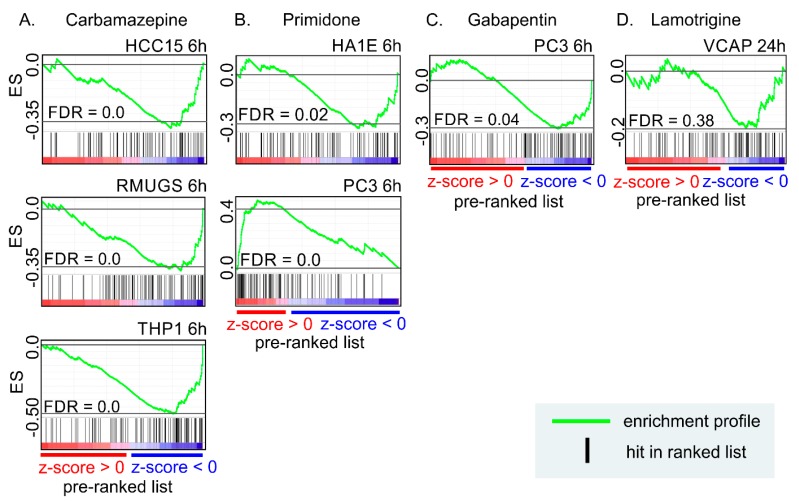
The effect of four anti-seizure drugs on EV-related genes in cell lines. The gene expression profiles of the anti-seizure drugs were downloaded from the LINCS database and used to prepare a rank lists for the Gene Set Enrichment Analysis. The top100 EV-related proteins from Vesiclepedia (http://microvesicles.org/, accessed on: 1 December 2018) were used as a gene set. (**A**) EV-related genes were negatively enriched by carbamazepine in the HCC, RMUGS, and THP1 cell lines. (**B**) Primidone treatment induced a negative enrichment in the HA1E cell line and a positive enrichment in the PC3 cell line. (**C**) Gabapentin treatment induced a negative enrichment. (**D**) Lamotrigine treatment did not show any enrichment of genes. ES, enrichment score; FDR, false discovery rate; HCC, Hepatocellular carcinoma cell line; HA1E, Cellosaurus cell line; PC3, human prostate cancer cell line; VCAP, human prostate cancer cell line; RMUGS, Cellosaurus cell line; THP1, human leukemic monocyte cell line.

Carbamazepine showed a negative enrichment in all cell lines (HCC15, RMUGS and THP1), indicating decreased expression of EV-related genes (Figure 3A). Primidone showed negative enrichment in Cellosaurus cell line (HA1E) and positive enrichment in human prostate cancer cell line (PC3) (Figure 3B). Gabapentin was also negatively enriched (Figure 3C). Lamotrigine did not show any significant enrichment (Figure 3D).

**Figure 4:**
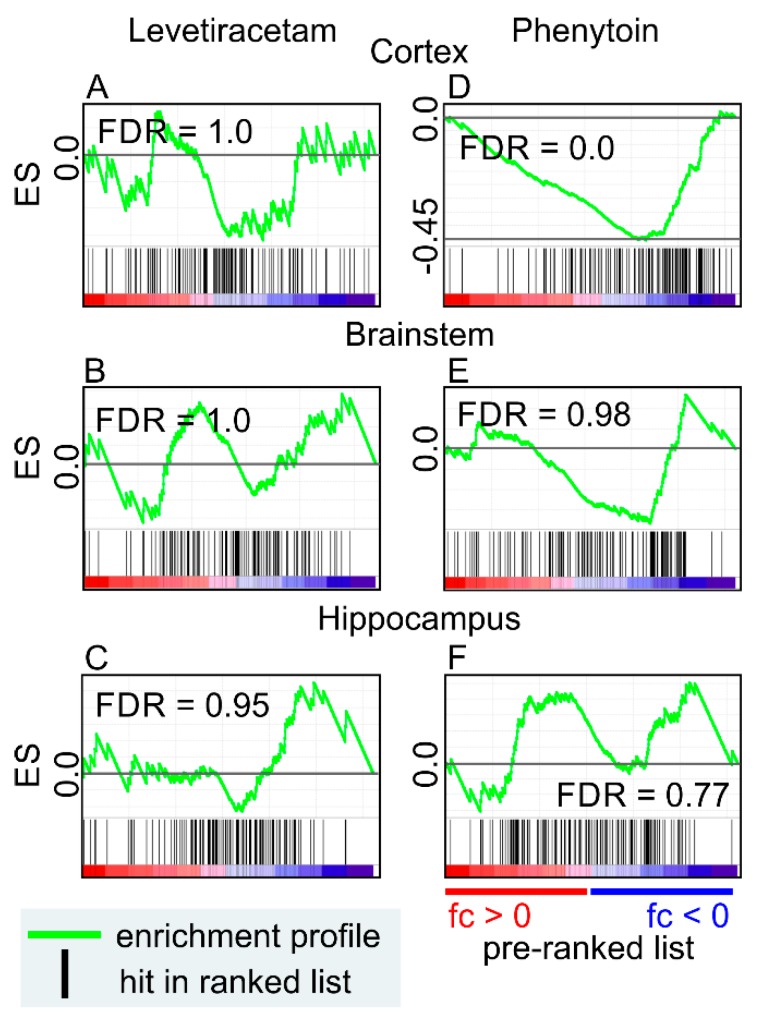
The effect of two antiepileptic drugs on EV-related genes in the cortex, brainstem, and hippocampus. Microarray gene expression data obtained from the rat brain tissue after a 90-d treatment with levetiracetam or phenytoin was used to construct a rank list for the Gene Set Enrichment Analysis. The top100 EV-related proteins from Vesiclepedia (http://microvesicles.org/, accessed on: 1 December 2018) were used as a gene set. (**A**–**C**) Levetiracetam treatment did not induce any enrichment of the EV-related genes in any of the brain areas. (**D**) Phenytoin treatment induced a negative enrichment of EV-related genes in the cortex. The effect was brain-region specific as no enrichment was observed in the brainstem or hippocampus (**E**,**F**). Abbreviations: ES, enrichment score; FDR, false discovery rate; fc, fold change.

Next, we investigated whether antiepileptic drugs modify EV-related gene expression also in vivo. We used phenytoin and levetiracetam-induced transcriptomic datasets of the rat cerebral cortex, brainstem, and hippocampus (GEO: GSE2880) [111]. By using GEO2R [112,113], we generated a gene list containing the fold-change (treatment vs control) of individual microarray probes. It was used as a rank list, and genes representing the top 100 EV-related proteins were used as a gene set in the GSEA. Levetiracetam did not show any significant enrichment in any of the studied brain areas (Figure 4A). EV-related proteins were negatively enriched in the cortex after phenytoin treatment, indicating that the drug might decrease EV production (Figure 4B). Brainstem and hippocampus did not show any significant enrichment by phenytoin treatment.

Our in silico analyses suggests that carbamazepine, primidone, and gabapentin can modify the EV-related gene expression in vitro, and phenytoin in vivo. In most cases, the gene expression was decreased. Interestingly, our literature analysis indicated an increase in the expression of EV-related genes in epileptogenesis and epilepsy in several rodent models. Further studies are needed to explore whether the modification of epileptogenesis-related EVs contributes to the mechanisms of actions of anti-seizure or antiepileptogenic drugs.

## 10. Conclusions

Extracellular vesicles show promise as diagnostic and prognostic biomarkers for epileptogenesis and epilepsy as several studies have reported acute changes in EV numbers and their cargo after epileptogenic brain insults such as SE or TBI. Our in silico analysis suggested that post-injury modulation of EV-related gene expression could be long-lasting. Importantly, EVs are also emerging as therapeutic agents to improve recovery such as SE and TBI. However, understanding the mechanistic and therapeutic potential of EVs in epileptogenesis and epilepsy and the effect of anti-seizure drugs on EV metabolism and cargo are in their infancy. Further studies are urgently needed to explore the potential of EVs as antiepileptogenic and co-morbidity-modifying agents as well as targets for treatment to improve the prognosis of epilepsy and those who are at risk of developing epilepsy after brain insults.

## Figures and Tables

**Table 1:** Articles describing the changes in extracellular vesicle (EV) number in epilepsy and traumatic brain injury (TBI).

Reference	Species	Disease	Tissue	Isolation Method	Analysis Method	Time Point (post-TBI)	Further EV Characterization	Results
[62]	human	partial epilepsy	CSF	10,000 g + 200,000 g ultracentrifugation	quantitative immunoblotting	no	no	Larger amount of CD133 on membrane particles in CSF of epilepsy patients
[72]	human	severe TBI	CSF	1500 g + 12,000 g centrifugation	functional prothrombinase assay	0 d, 3 d, 5 d, 10 d	no	EV number at day 0 post-TBI clearly higher than in control group, but decreased towards day 10
[72]	human	severe TBI	plasma	1500 g + 12,000 g centrifugation	functional prothrombinase assay	0 d, 3 d, 5 d, 10 d	no	EV number at day 0 post-TBI higher than in controls, decreased progressively between day 0 and day 10, at day 10 about same level as controls
[73]	human	severe TBI	plasma (arterial)	2000 g + 13,000 g centrifugation	flow cytometry	emergency room, 6 h, 12 h, 24 h, 2 d, 3 d	no	EV number highest at the emergency room, decreased during the 3 days post-TBI
[73]	human	severe TBI	plasma (cerebrovenous)	2000 g + 13,000 g centrifugation	flow cytometry	6 h, 12 h, 24 h, 2 d, 3 d	no	EV number increased after TBI, decreases during the 3 days post-TBI
[74]	human	TBI	CSF	170,000 g ultracentrifugation	flow cytometry	after TBI	EM and fluorescence microscopy	Increased number of EVs post-TBI
[75]	human	severe TBI	CSF	2 × 100,000 g ultracentrifugation	NTA	12 h	TEM, Western blot	Increased EV concentration and smaller EVs after TBI
[76]	human	severe TBI	CSF	2 × 100,000 g ultracentrifugation	NTA	d 1, d 2–3, d 4–7	TEM, Western blot	Highest EV concentration 24 h after injury. Larger EVs on days 4-7 post-TBI
[66]	mouse	TBI	plasma	ExoQuick kit	flow cytometry	24 h	EM	Increased EV number after TBI
[67]	mouse	TBI	total blood	1500 g + 15,000 g + 100,000 g ultracentrifugation	flow cytometry	24 h	no	Increased number of total blood EVs and microglial EVs after TBI
[68]	mouse	TBI	plasma	120,000 g ultracentrifugation	TRPS	2 h, 6 h, 12 h, 24 h	TEM, Western blot	Increased EV concentration 24 h after injury. Smaller vesicles at 24 h post-TBI
[71]	mouse	TBI	plasma	10,000 g centrifugation	NTA	30 min, 3 h, 24 h, 3 d	no	Decreased EV number 3 h and 24 h after injury, returned to sham levels by 3 d post-TBI. The share of platelet-derived (CD41+) EVs increased from 3 h to 24 h.
[69]	piglet	TBI	serum	2300 g centrifugation	flow cytometry	before and after TBI	no	EV number is increased post-TBI
[70]	rat	TBI	brain tissue	no isolation	quantitative immunoblotting	2–8 h	Western blot	Increased expression of CD63 and CD81 in hippocampal EVs at 6 h post-injury.

Abbreviations: CSF = cerebrospinal fluid, NTA = nanoparticle tracking analysis, EM = electron microscopy, TEM = transmission electron microscopy, TRPS = tunable resistive pulse sensing

**Table 2:** Articles describing the beneficial effect of extracellular vesicle (EV) therapy in status epilepticus (SE) and traumatic brain injury (TBI) models.

Article	Species	Model	Dose and Time Point	EV Type	What was Measured	Isolation Method	Characterization of EVs	Main Finding
[82]	rat	controlled cortical impact -induced TBI	100 µg total proteins, 1 d post-injury	rat MSC EVs	Foot-Fault Test, modified Morris water maze, modified Neurological Severity Score, immunohistochemistry	ExoQuick	Total protein concentration, qNano	EVs improved spatial learning and sensorimotor functional recovery, reduced neuroinflammation and increased the number of newly generated endothelial cells.
[83]	rat	controlled cortical impact -induced TBI	100 µg proteins, 3 × 10^9^ particles, 1 d after injury	human MSC EVs, cultured in 2D and 3D conditions	Modified neurological severity score, foot-fault test, Morris water maze, immunohistochemistry	ExoQuick	Total protein concentration, qNano	EVs enhanced spatial learning, reduced brain inflammation, increased neurogenesis in DG, vascular density and angiogenesis
[84]	rat	free -falling method	100, 250, 500 and 1000 µg/mL, time not mentioned	human exfoliated deciduous teeth stem cell EVs	Basso, Beattie and Bresnahan scores, histopathology and immunofluorescense	ExoQuick	Flow cytometry with CD81, CD63 and CD9, TEM, Western blot with CD9 and CD63	EVs improved rat motor functional recovery and reduced cortical lesion 2 weeks post-injury
[85]	mouse	1 h post-TBI	human MSC EVs	human MSC EVs	Morris water maze, pattern separation test, immunohistochemistry, cytokines in plasma	Anion exchange column	NTA	EVs rescued pattern separation and spatial learning impairments
[86]	swine	computer-controlled cortical impact -induced TBI	1 × 10^13^ particles, 9 h, 1 d, 5 d, 9 d, and 13 d post-injury	human MSC EVs	Neurocognitive function test, neurologic severity score (NSS)	Sequential centrifugation	qNano	EV treated animals had better neurological functions first 5 d post-TBI and they completed neurological recovery in shorter time
[87]	mouse	controlled cortical impact -induced TBI	EVs from 4 × 10^6^ cells, 2 h post-TBI	endothelial colony-forming cell EVs	Brain water content, beam-walking, corner test, immunofluorescence	Sequential centrifugation	TEM, NTA and Western blot with CD9, CD81 and HSP70	EVs inhibited PTEN expression, increased AKT expression and reduced Evans blue dye extravasation, brain edema and tight junction degradation
[88]	rat	mild controlled cortical impact -induced TBI	100 µg total proteins, 3 h post-TBI	adipose-derived stem cell EVs	Elevated body swing test, forelimb akinesia, paw grasp, in vivo and ex vivo imaging, immunohistochemistry and RNA sequencing	ExoQuick following magnetic bead capture with CD9, CD63 and CD81	NTA	MALAT1 containing EVs promoted recovery of function on motor behavior and reduction in cortical brain injury
[89]	mouse	pilocarpine-induced SE	30 µg, approximately 15x10^9 particles, same day and 18 h after SE	human MSC from bone marrow EVs	Object location test, novel object recognition test, pattern separation test, immunostaining, cytokine levels	Anion exchange column	Protein concentration, NTA, anti-inflammatory assay	EVs reduced inflammation in hippocampus, repressed neurodegeneration, aberrant neurogenesis and cognitive and memory impairments

Abbreviations: MSC = mesenchymal stem cell, NTA = nanoparticle tracking analysis, DG = dentate gyrus, TEM = transmission electron microscopy

**Table 3:** Articles describing the changes in extracellular vesicle (EV) cargo RNA in status epilepticus (SE) and traumatic brain injury (TBI).

Article	Species	Condition	Starting Material	Isolation Method	Characterization of EVs	What was Measured	Main Finding
[58]	mouse and human	blast overexposure injury (mice) and TBI patients	plasma and serum	microchip using GluR2 antibody	DLS and SEM in method set-up with CCM	miRNA-seq, 7 miRNAs validated	miRNA-based biomarker panel for diagnosis of TBI
[63]	human	TLE and SE patients	CSF	ExoQuick reagent	no characterization	miR-19b-3p, miR-21-5p and miR-451a (top findings)	EV-cargo miRNAs showed more promise than Argonaute2 bound miRNAs as biomarkers
[64]	human	TLE patients	plasma	ExoQuick reagent	no characterization	miR-27a, miR-328-3p and miR-654-3p levels (top findings)	Higher diagnostic accuracy with EV-cargo miRNAs as compared to Argonaute2 bound miRNAs
[65]	human	mTLE-HS patients	plasma	ExoQuick reagent	TEM or Western blot (not shown)	microarray	50 differentially expressed miRNAs, 6 validated (miR-3613-5p, miR-4668-5p, miR8071, miR-197-5p, miR-4322 and miR-6781-5p)
[74]	human	severe TBI	CSF	ultracentrifugation	TEM, flow cytometry	microarray	81 miRNAs found, miR-9 and miR-451 differentially packed after TBI
[80]	mouse	controlled cortical impact-induced TBI	brain tissue	digestion of brain tissue and ultracentrifugation	TEM	miRNA-seq	miR-212 decreased and miR-21, miR-146, miR-7a, and miR-7b increased
[81]	mouse	fluid percussion-induced TBI	extracellular space	digestion of brain tissue and Total Exosome Isolation reagent	TEM, Western blot	circ-RNA-seq	231 differentially expressed circular RNAs, 5 validated

Abbreviations: TLE = temporal lobe epilepsy, CSF = cerebrospinal fluid, TEM = transmission electron microscopy, DLS = dynamic light scattering, SEM = scanning electron microscopy, CCM = cell culture media.
